# Pet food choices in transition: how owner demographics and diets influence pet food selection and the acceptance of alternative protein sources in pet feeding

**DOI:** 10.3389/fvets.2026.1836864

**Published:** 2026-05-15

**Authors:** Ammelie Godglück, Bettina Schneider, Christian Visscher, Volker Wilke

**Affiliations:** 1 Institute for Animal Nutrition, University of Veterinary Medicine Hannover, Hannover, Germany; 2 Institute of Biometry, Epidemiology and Information Processing, University of Veterinary Medicine Hannover, Hannover, Germany

**Keywords:** alternative protein sources, dog and cat nutrition, human-animal interaction, owner consuming behavior, pet feeding practices

## Abstract

Given the increasing interest in alternative protein sources and growing ethical awareness in nutrition, this large-scale survey (*n* = 8,823) investigated the feeding practices of dog and cat owners in mainly German-speaking countries. The aim was to assess preferences, feeding habits, and the acceptance of novel protein sources such as plant- and insect-based pet foods. The results showed that conventional meat-based diets remained predominant (i.e., 53.05% daily feeding of meat-based dry food in dogs), but there was notable openness toward alternatives, especially among younger female and vegan pet owners. Acceptance of vegan diets was higher for dog owners than for cat owners (53.9% of vegan dog owners also fed their dogs a vegan diet compared to only 10% of vegan cat owners), which may reflect differences in perceived species-specific nutritional requirements. Insect-based diets were used less frequently but were perceived by more respondents as biologically appropriate compared to vegan diets. The main driver for diet selection was pet health, while sustainability played a secondary role. Veterinary advice was cited by only 14.11% of owners, with online sources and peer recommendations being more influential. Overall, the findings highlight how owner demographics, personal dietary identity, and perceived nutritional suitability shape decision-making in pet feeding and influence the acceptance of alternative protein sources.

## 1 Introduction

In the coevolution between dogs and humans, dogs fulfilled various tasks, from hunting to herding to emotionally important, fully fledged family members (1). There is a close connection between owners and their dogs, and it has been shown that interactions increase oxytocin secretion in both humans and dogs (2). Therefore, given the close relationships between dogs and their owners it is not surprising that the human way of life and human nutrition have a huge influence on how pet owners feed their dogs (3). In recent years, people have become increasingly interested in vegan and vegetarian diets; globally, the diets which were searched the most by Google users were veganism and vegetarianism (4).

Increasingly more people are choosing a vegetarian or vegan lifestyle for ethical, health, and environmental reasons (5). Again, there are demographic and geographic differences: most vegetarians and vegans are female, under 30 years old, have a higher level of education, and tend to live in larger cities. Nevertheless, a vegetarian and vegan lifestyle remains present across all age groups, with a slightly lower proportions among individuals aged 30 years and older (6, 7). Veganism has become more of a lifestyle than a way of eating and is motivated by various reasons, including becoming a social movement (8). Many vegans own pets, and it is not uncommon for these people to face a moral dilemma when it comes to feeding their dogs and cats, especially if the owners themselves have chosen to go vegan for ethical reasons (9). Therefore, there are mainly vegan or vegetarian pet owners who are interested in feeding their pets a plant-based diet, or who are already feeding their pets a plant-based diet (10).

In parallel with plant-based protein sources, insects are another new source of protein (11). Various studies show that insect protein can also be a possible alternative to common meat-based protein sources (12–14). Insects are being used as a source of protein in the pet food industry, but attention must be paid to possible allergies and intolerances (15). In human nutrition, it remains to be seen at European level whether insects can establish themselves as a long-term source of protein and be accepted by consumers (16).

Dog owners consider good nutrition to be a very important factor in the well-being of their dogs. In fact, in a questionnaire where dog owners were asked what the most important factors for a dog's well-being were, over 50% stated a good diet, while a regular veterinary check was ranked as the least important one (17). However, many pet owners receive their nutritional advice from friends and family or via the internet rather than from their veterinarian (18). Even within the veterinary profession, there remains room for improvement in the field of nutritional counseling; while therapeutic diets are frequently offered, comprehensive nutritional consultations are not always provided (19).

Pets that are fed a meat-based diet, such as dogs and cats, have a higher ecological footprint than those that are fed a plant-based diet, such as rabbits and birds, mainly due to the nature of their diet (20). A dog produces around 630 kg CO_2_ equivalents per year, which is equivalent to around 7% of the emissions of a German citizen, and pet food is a major contributor, especially the ingredients and the packaging (21). In general, animal-based ingredients have a higher carbon footprint than plant-based ingredients, with ruminant meat having the greatest impact per kilogram, both in terms of greenhouse gas emissions and land use (22). On a global scale, Alexander et al. (23) estimated by using economic allocation that pet food is responsible for 1.1 to 2.9% of global greenhouse gas emissions from agriculture and 0.8 to 1.2% of global agricultural land use; the higher the protein and meat content of pet food, the higher the greenhouse gas emissions and agricultural land use. Other models using mass allocation yield higher results, showing that animal byproducts often have lower protein density and can require more livestock carcasses to provide the same amount of protein (24, 25). The environmental impact of pet food is strongly driven by ingredient selection, particularly the level and type of protein, while packaging and manufacturing play a comparatively minor role (24, 26).

## 2 Material and methods

The survey was designed to evaluate the feeding practices of pet owners and to explore the interest of pet owners in novel protein sources like insects or plant-based protein in pet food. It was conducted via an online survey tool (LimeSurvey GmbH, Hamburg, Germany) and was available in English and German. The estimated completion time for the pet owners ranged from 6 to 8 minutes. Before answering the questionnaire, the respondents were informed about the aims of the study and the data protection agreement. The questionnaire included 40 questions, subdivided into five sections: (A) demographics and dietary habits of owners, (B) specific pet information, (C) dietary habits of pets, (D) choice of food, and (E) alternative protein sources. The survey was distributed via social media channels of the University of Veterinary Medicine Hannover and was openly accessible for further dissemination to reach a broad audience of pet owners. It was primarily shared by dog related accounts. The survey was not specifically targeted to particular groups or forums, and participation was voluntary without incentives, representing a convenience sample. The survey was online from August 15, 2023 to December 15, 2023. The entire survey is included in the Appendix. There were no formal restrictions for participation, however respondents were required to be pet owners. In cases where respondents owned multiple animals, they were instructed to complete one questionnaire per animal.

Group A questions focused on the owners, including demographic information like place of residence, gender, age, and behavior such as own dietary habits using a single-choice item. Group B questions asked about specific pet information. The respondents were asked to state their pets' species (dog or cat), the breed, age, weight, sex, and any known allergies. Information on food intolerances or allergies was based on owner-reported data. Respondents could indicate whether no intolerance was present, whether an intolerance was suspected without diagnostic confirmation, or whether it had been diagnosed either by blood testing or by an elimination diet. No independent verification of these reports was performed. The questions about weight and age were free text answers, the other questions were single choice questions. The third section of the questionnaire (C) comprised items related to dietary feeding habits. The questions included both single- and multiple-choice questions. In this section the type of feeding, the duration of the current feeding, and the protein and carbohydrate sources of the current feed were recorded. There were also questions about the feeding of snacks and treats. The following section (D) included questions about the choice of pet food. Here, too, there were predominantly multiple-choice questions in addition to five-point Likert scale questions. The multiple-choice questions mainly dealt with the reasons for deciding to buy and feed a particular pet food. The questions on the five-point Likert scale ranging from 1 = strongly agree to 5 = strongly disagree, mainly related to the subjective quality characteristics of certain feedstuffs and the overall assessment of the quality of pet food. The final section (E) included questions on alternative protein sources. There were predominantly single-choice questions in addition to five-point Likert scale questions ranging from 1 = strongly agree to 5 = strongly disagree.

The dataset comprised 11,248 questionnaires. Incomplete submissions with less than 25% and cancellations were excluded. A total of 8,823 questionnaires met the inclusion criteria and underwent statistical analysis. All statistical analyses were conducted using SAS^®^ software (Version 9.4, SAS Institute Inc., Cary, NC, USA). A descriptive statistical analysis was performed to summarize demographic variables and feeding practices. Results were expressed as absolute and relative frequencies. To assess associations between categorical variables, Chi-square tests were applied. Statistical significance was set at *p* < 0.05.

## 3 Results

### 3.1 Demographics and own dietary habits

Of the participants, 94.3% (8,320) were dog owners, 5.7% (503) were cat owners, 89.91% (7,933) of the participants lived in Germany, 3.9% (344) in Switzerland, 4.0% (353) in Austria, and 2.19% (193) in non-German speaking countries. The majority of owners were female (93.74%; 8,271), while 4.75 % (419) were male. A total of 0.35% (31) described themselves as diverse and 1.15% (102) did not specify their gender. The highest group of owners were aged between 30 and 60 years (76.05%; 6,710), a smaller group was under 30 years old (13.14%; 1,159) or over 60 years old (10.3%; 909). When being asked about their own dietary habits; a total of 9.49 % (837) pet owners responded that they were vegan, 14.94% (1,318) answered that they were vegetarian, and 73.67% (6,500) that they were omnivorous; a total of 1.9 % of the respondents did not provide any information.

### 3.2 Specific pet information

The median age of the dogs was 5.08 years, with a standard deviation of 4.09 years. For the cats, the median age was 8.00 years, with a standard deviation of 5.04 years. The median weight of the dogs was 21.00 kg, with a standard deviation of 11.19 kg, while the cats had a median weight of 5.00 kg and a standard deviation of 3.82 kg. Of the dogs, 22.55% were male, 27.03% were neutered males, 20.69% were female, and 29.53% were neutered females. Of the cats, 2.58% were male, 57.06% were neutered males, 0.99% were female, and 39.17% were neutered females. A total of 65.63% of the dogs and 80.72% of the cats showed no signs of food intolerance. Suspected food intolerance was noted in 16.07% of the dogs and 9.74% of the cats. In 6.01% of the dogs and 2.58% of the cats, food intolerance had been confirmed through blood testing, while 11.86% of the dogs and 6.96% of the cats had had food intolerance confirmed via an exclusion diet.

### 3.3 Dietary feeding habits

**Figure 1:**
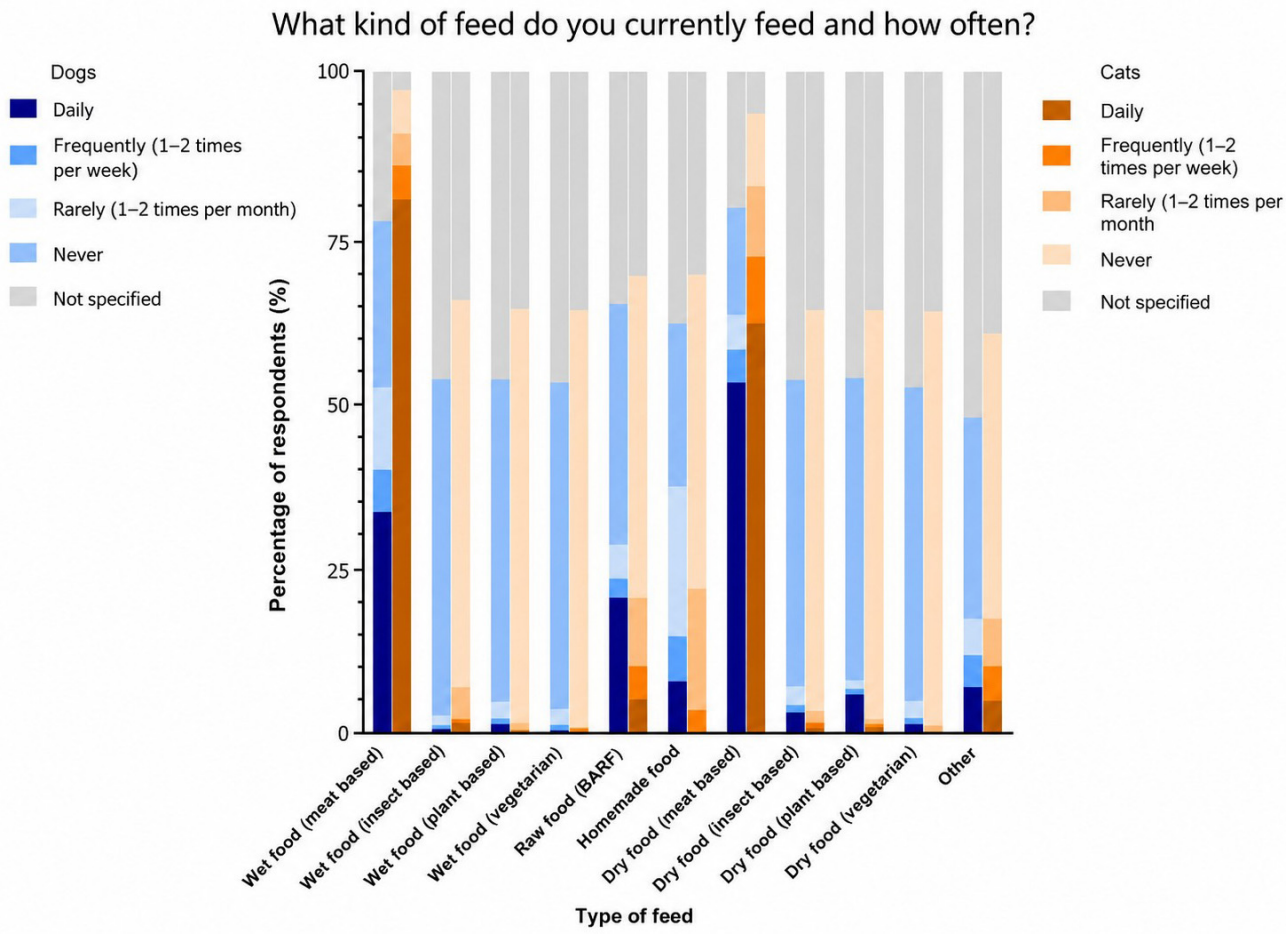
Descriptive analysis of feeding frequency across various dog and cat food types in %. BARF, biologically appropriate raw food.

Figure 1 presents a descriptive analysis of feeding frequency across various dog and cat food types. The food types include wet food (meat-based, insect-based, vegan, vegetarian), biologically appropriate raw food (BARF), homemade food, other types, and dry food (meat-based, insect-based, vegan, vegetarian). Responses were categorized into four feeding frequencies: “Daily”, “Frequently” (1–2 times per week), “Rarely” (1–2 times per month), and “Never”. Overall, for dogs, the most common feeding type was meat-based dry food (53.03% daily feeding), followed by meat-based wet food (33.56% daily feeding). For the alternative protein sources, plant-based dry food (5.91% daily feeding) was most common, followed by insect-based dry food (3.17% daily feeding). For cats, the most common feeding type was meat-based wet food (80.72% daily feeding), followed by meat-based dry food (62.03% daily feeding). The most common cat food for alternative protein sources was insect-based wet food (1.59% daily feeding).

Pet owners were surveyed regarding the primary protein source in their pets' diet. For dogs, the predominant protein source was meat and slaughter by-products (82.24%), followed by plant-based proteins (7.91%), fish (5.70%), and insect-based proteins (2.90%). For cats, the main protein source was meat and slaughter by-products (87.48%), followed by fish (8.95%), plant-based proteins (1.79%), and insect-based proteins (1.39%). The most common used carbohydrate sources for dogs were potatoes (30.31%), followed by rice (21.05%) and sweet potatoes (15.07%). The most common used carbohydrate sources for cats were rice (15.51%), followed by wheat (14.31%) and potatoes (10.93%).

When pet owners were asked about the frequency of snack feeding, 49.39% of dog owners said they fed snacks several times a day, while 20.87% of the cat owners responded that they fed snacks several times a day. A total of 2.19% of dog owners and 7.55% of cat owners stated that they never fed any snacks. The most common snacks for dogs (67.37%) and cats (84.3%) were meat-based snacks, followed by a combination of vegetarian and meat-based snacks given by 8.10% of the dog owners and 3.5% of the cat owners.

### 3.4 Food choice

**Figure 2:**
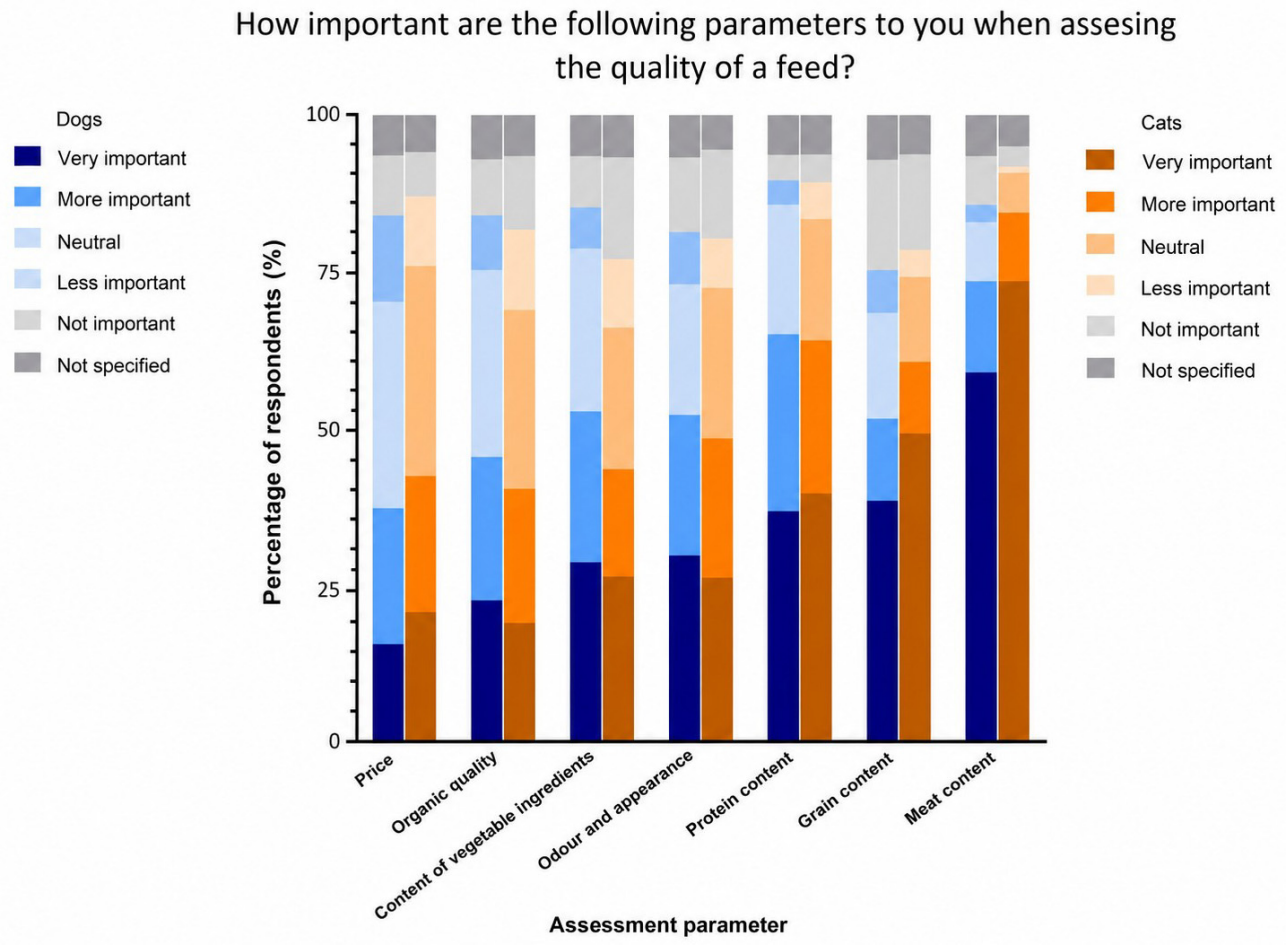
Quality criteria for pet food assessed by the owners in %. (Specifications regarding grain content can be found in Figure 3).

When asked about the reasons for selecting their pets' current diet, 22.95% of dog and cat owners reported relying on information obtained from the internet. Veterinary advice influenced the decision of 14.11% of pet owners, while 10.63% based their choice on the information provided on the packaging. Additionally, 28.61% of owners made their decision based on recommendations from other pet owners, breeders, specialist retailers, or print media. A further 23.10% indicated that they obtained such information from other sources. Pet owners were most likely to buy their pet food online (36%), followed by pet shops (15.28%) and directly from the producer (7.05%). A total of 1.11% of pet owners bought their pet food from the veterinarian. When selecting the food, the health of their pets was most important to both dog (78.4 %) and cat (78.73%) owners. In contrast, the sustainability of the pet food was most frequently rated as less important (dogs: 13.08%; cats: 17.89%). When assessing the quality of a pet food (Figure 2), 56.36% of dog owners and 71.17% of cat owners stated that the meat content was the most important factor, followed by the grain (dogs: 34.98%; cats: 46.12%) and protein (dogs: 33.35%; cats: 36.18%) content. At the same time, 19.04% of dog owners rated the grain content as unimportant, followed by the odor and appearance of the food (12.79%). The proportion of vegetable components was least important to cat owners (17.69%), followed by grain content (16.50%) and odor and appearance (15.31%).

**Figure 3:**
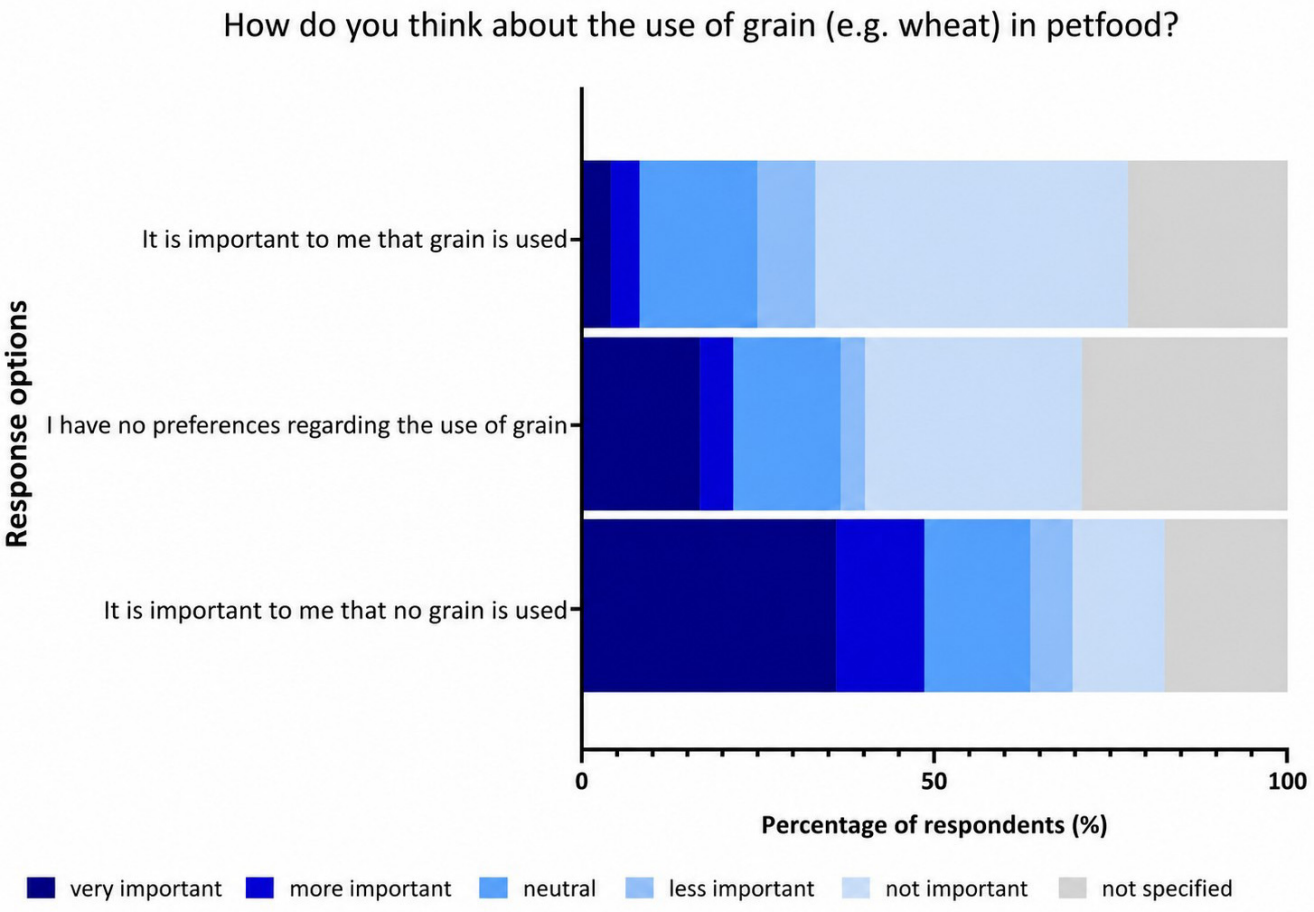
Responses of dog owners regarding the importance of grain in pet food in %.

A high meat content in pet food was considered important by 47.75% of dog owners and 67.20% of cat owners. According to the pet owners surveyed, this should be made up of a high proportion of lean meat (dogs: 20.66%; cats: 29.42%) and without any animal by-products (dogs: 27.38%; cats: 23.46%).

For most dog owners, it was very important that no grain was used in dog food (36.12%). A total of 16.74% of dog owners had no preference regarding the use of grain in dog food, while 4.07% of the owners considered it important.

### 3.5 Alternative protein sources

**Figure 4:**
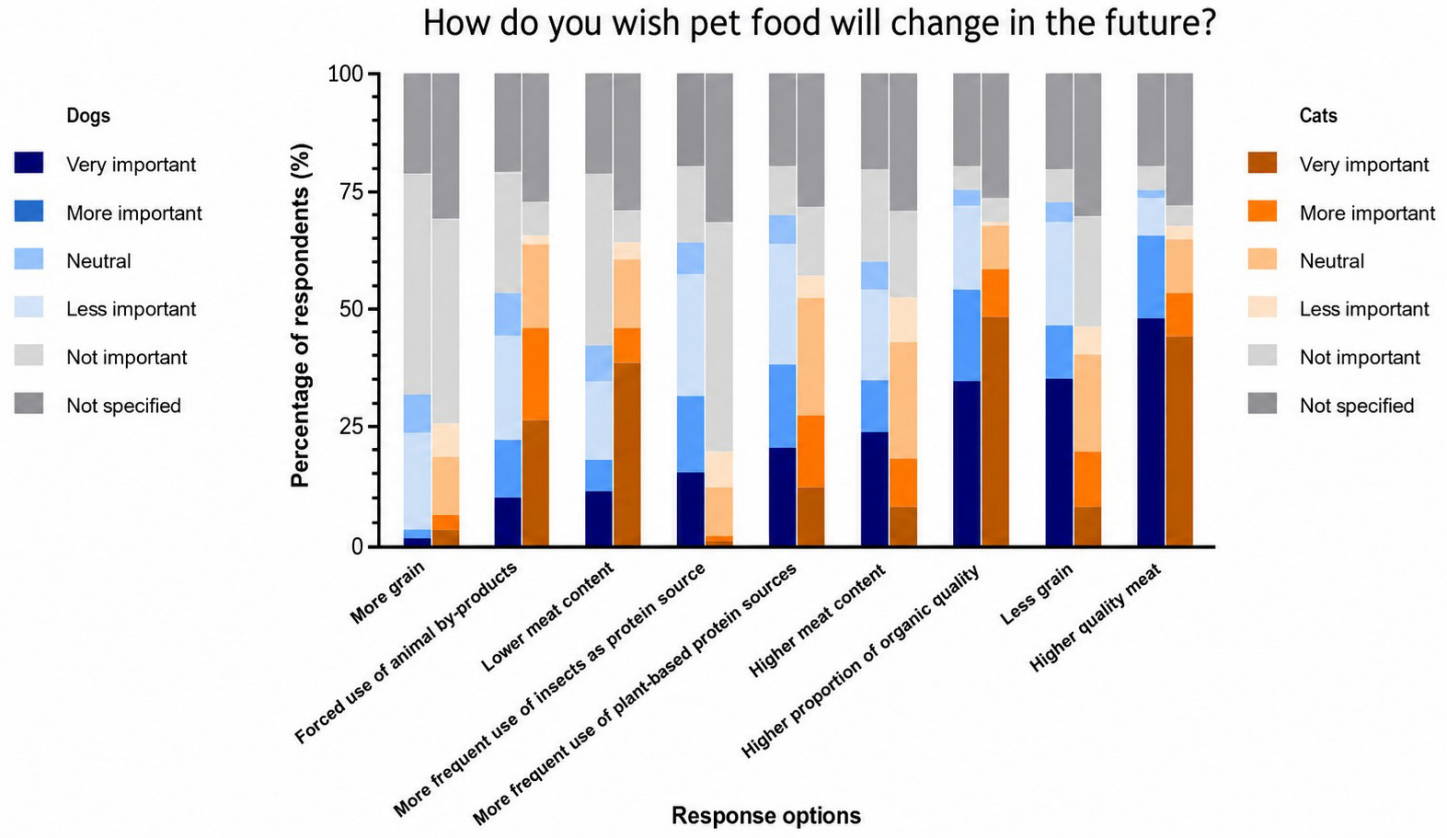
How dog owners envision the future of dog feeding in %.

Of the surveyed owners, 53.64% of dog owners and 67.79% of cat owners considered meat-based diets to be *species-appropriate*, with 27.40% of dog owners and 42.15% of cat owners believing such diets *provide all essential nutrients*. Only 7.49% of dog owners and 2.98% of cat owners regarded vegan diets as *species-appropriate*, while 9.90% of dog owners and 8.55% of cat owners considered insect-based diets *suitable* for their pets. A total of 27.80% of dog owners and 35.19% of cat owners perceived vegan diets as *potentially harmful* to their pets' health. When asked whether they would feed their animals a vegan food, 48.53% of dog owners and 59.24% of cat owners answered no. In the case of an insect-based pet food, 25.66% of dog owners and 20.08% of cat owners ruled out feeding it to their pets. Reasons for buying vegan food for dog and cat owners would mainly be the health of their pet (dogs: 54.82%; cats: 44.73%) and intolerances to other protein sources (dogs: 54.18%; cats: 43.94%). The pet owners surveyed also gave the same reasons for buying insect-based pet food. Figure 4 shows how pet owners would like to see pet food develop in the future.

A total of 46.85% dog owners stated that they would like higher quality meat to be used in their dog's food. In addition, 34.81% said that the inclusion of less grain in dog food was also very important.

Cat owners, on the other hand, considered organic quality (46.92%) and generally higher meat quality (42.74%) to be most important, whereas less grain (9.94%) in cat food only played a minor role.

### 3.6 Influence of owner demographics and dietary habits on pet feeding practices

In a second step, the effects of the demographic data of the pets and their owners as well as the owners' own eating habits on feeding behavior were analyzed. It was shown that the gender of the pet owner had a significant influence on the choice of protein source. Among 404 male dog owners, 14.11% chose a vegan dog food, while 7.45% of the 7,792 female dog owners chose a vegan food. Of the 31 dog owners who stated their gender as diverse, 41.94% fed their pets vegan food. There were no significant differences among cat owners in terms of the gender of the owner and the protein source used.

**Figure 5:**
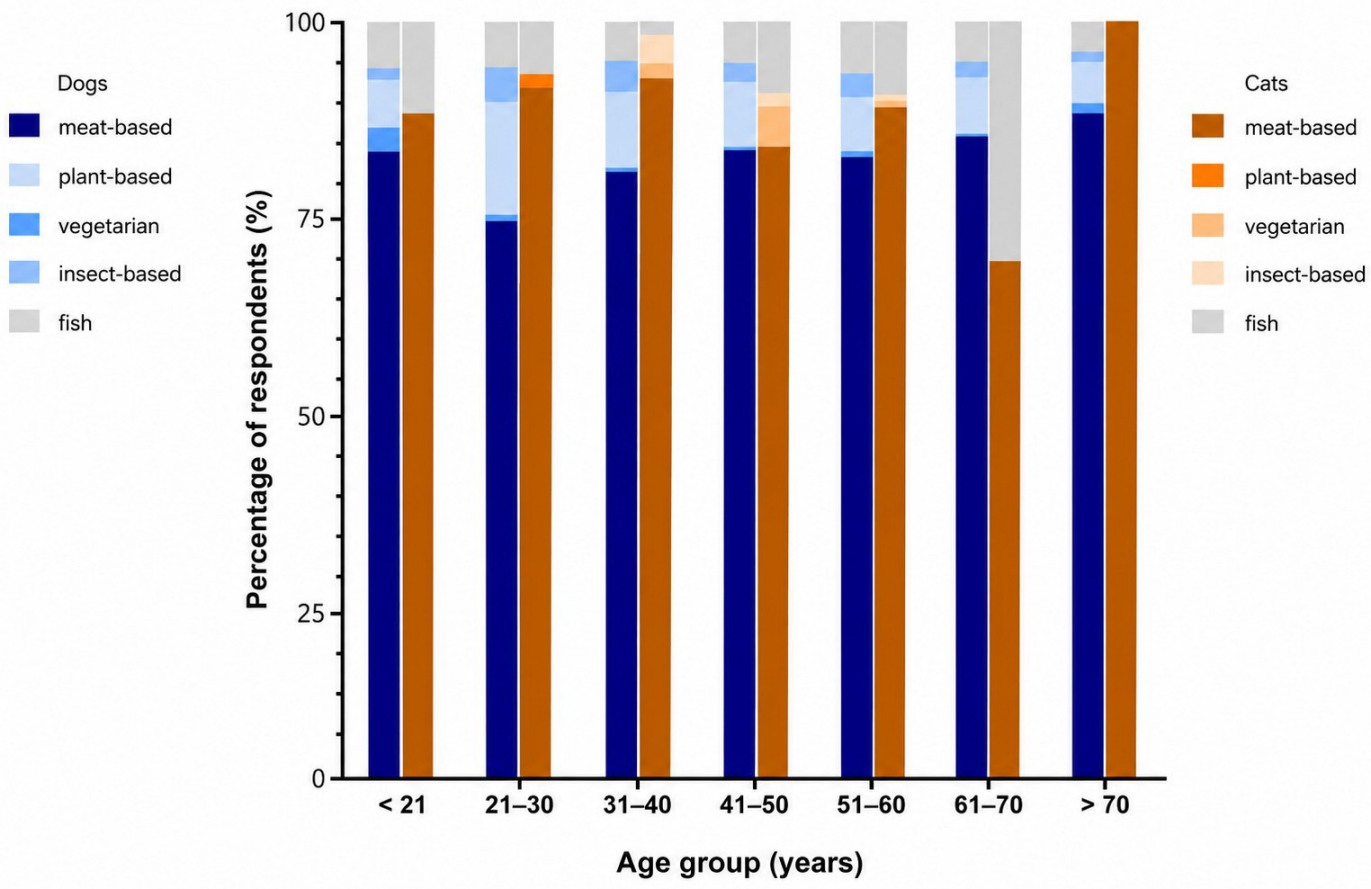
Influence of pet owners' age on the choice of protein sources used in dog and cat diets in % (dogs: *p* < 0.0001, Cramer's V (*V*) = 0.0552; cats: *p* = 0.0021, *V* = 0.1588).

The influence of the dog owner's age on the choice of protein source was also significantly different (Figure 5). The 21–30 age group most frequently opted for plant-based (13.23%) or insect-based (4.24%) protein sources. In contrast, the age group over 70 was the least likely to feed plant-based (4.94%) or insect- based (1.23%) protein sources. There were also significant differences in the choice of protein source between the different age groups of cat owners. Here, the age group between 61 and 70 years used meat as a protein source the least (71.43%), while the proportion of fish as a protein source was highest here with 28.57 %.

**Figure 6:**
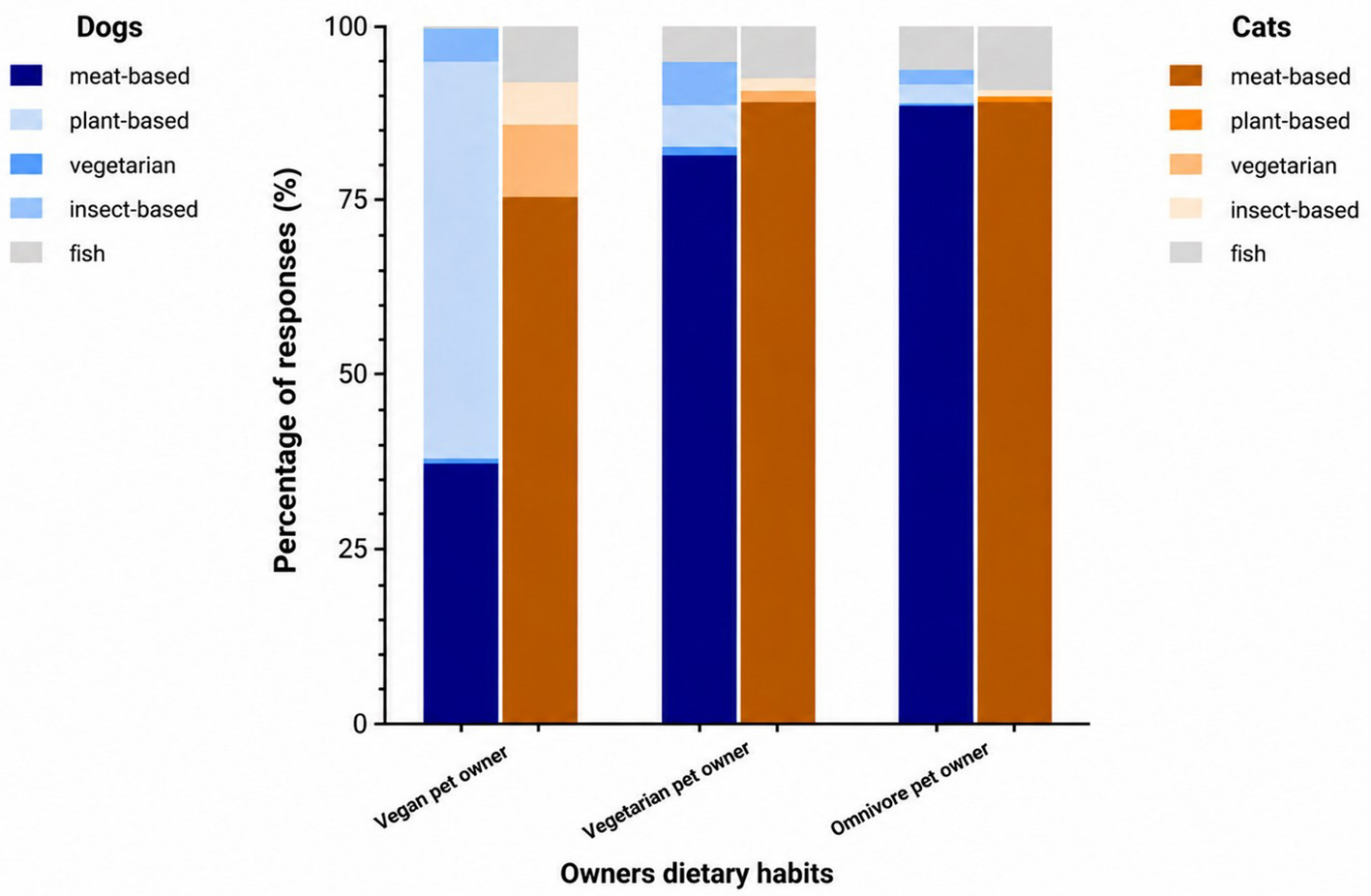
Influence of pet owners' dietary habits on the choice of protein sources in dog and cat diets in % (dogs: *p* < 0.0001, *V* = 0.3998; cats: *p* < 0.0001, *V* = 0.1856).

It was observed that vegan dog owners fed their dogs a vegan diet significantly more often than dog owners with other eating habits (Figure 6). Of the vegan dog owners, 53.90% also fed their dogs a purely plant-based diet, while 5.88% of the vegetarian and 2.53% of the omnivore dog owners fed their dogs a plant-based diet. The same trend could be observed among cat owners, although only 10.00% of vegan cat owners also fed their pets a plant-based diet, while 1.52% of the vegetarian and 0.55% of the omnivore cat owners fed their cats a plant-based diet.

When choosing meat-based food, vegan dog and cat owners considered animal welfare, sustainability, and organic meat production to be more important than pet owners who did not follow a vegan diet. Regarding animal welfare, 57.37% of vegan dog owners and 47.92% of vegan cat owners selected ‘strongly agree'. In contrast, only 19.98% of omnivore cat owners and 25.14% of omnivore dog owners rated the aspect of animal welfare as ‘strongly agree', a difference that was statistically significant (dogs: *p* < 0.0001, *V* = 0.1464; cats: *p* = 0.0023, *V* = 0.1622). The situation was similar concerning the question of sustainability of meat protein sources, with 48.15% of vegan dog owners and 39.58% of vegan cat owners rating this aspect with “*strongly agree*”, while 21.54% of omnivore dog owners and 15.79% of omnivore cat owners rated sustainability with “*strongly agree*” (dogs: *p* < 0.0001, *V* = 0.1235; cats: *p* = 0.0039, *V* = 0.1581). When assessing the quality of pet food, 47.45% of vegan dog owners rated the meat content as unimportant, whereas 66.42% of carnivore dog owners rated the meat content as very important (*p* < 0.0001, *V* = 0.3016). There were no significant differences between the different dietary habits of cat owners on this point.

A total of 77.91% of dogs diagnosed with food intolerance through an exclusion diet were fed a meat protein source, while 7.98% were fed a plant-based protein diet and 7.36% were fed an insect-based diet. Regarding cats, 88.57% of the food intolerant animals were fed a meat-based protein source. For the carbohydrate sources used, significantly (*p* < 0.0001, *V* = 0.1023) more sweet potatoes was used for dogs with a food intolerance (24.90%) than in those without a food intolerance (12.55%). This was not observed in cats.

## 4 Discussion

This large-scale survey provides valuable insights into the feeding practices of pet owners, with a particular focus on the acceptance and use of alternative protein sources such as plant-based and insect-based proteins. The high number of responses (*n* = 8,823) allows for a representative picture, particularly in German-speaking countries. Given the large sample size and the number of comparisons performed, statistically significant results may also reflect small differences of limited practical relevance. Therefore, the interpretation focusses on the magnitude and practical relevance of the observed effects, as reflected by effect size measures such as Cramers V, rather than statistical significance alone. Differences between dogs and cats must be interpreted in the light of their distinct nutritional physiology. While dogs are facultative carnivores and can adapt to a broader range of diets, cats are obligate carnivores (27, 28).

As expected, traditional meat-based diets remain the most common feeding practice among both dog and cat owners. The collected data illustrate distinct demographic patterns in the acceptance of alternative protein sources. For example, the age of the pet owner had a significant effect on the feeding decisions. In the group of dog owners aged 21–30 years, 13.23% opted for plant-based proteins and 4.24% for insect-based proteins, while these values dropped to 4.94% (plant-based) and 1.23% (insect-based) for owners older than 70. Among cat owners, significant age effects were also present: the group aged 61–70 years reported the lowest use of meat (71.43%) but the highest proportion of fish-based feeding (28.57%). These observations are consistent with findings from a survey on food consumption habits in Germany, which showed that 58% of respondents frequently purchased vegetarian or vegan substitute products compared with only 24% of individuals aged over 60 years; similar age-related patterns have also been reported in other studies, for example from North America, confirming this observation across different cultural contexts (6, 10, 29).

The owners' personal dietary habits, vegan, vegetarian or omnivorous, were closely linked to feeding behavior. More than half of vegan dog owners (53.90%) also fed their dogs a vegan diet compared to a much lower proportion among non-vegan owners. Among cat owners, the trend was similar but less pronounced, with only 10.00% of vegan owners giving their cats a vegan diet. This finding is consistent with Weber et al. who demonstrated that diet is closely linked to gender role self- concept and moral disengagement mechanisms (30). The discrepancy between the feeding of dogs and cats likely reflects the species-specific differences in nutritional physiology and the public perception thereof, direct comparisons between species should be interpreted with caution. While dogs are facultative carnivores and can adapt to a plant-based diet under certain conditions, cats are obligate carnivores with specific nutritional requirements that are difficult to meet through plant-based sources alone (27, 28). When vegan owners nevertheless selected meat-based foods, their decision was guided by ethical considerations: 57.37% of vegan dog owners and 47.92% of vegan cat owners rated animal welfare with an agreement level of “*strongly agree”* compared with only 25.14% of carnivore dog owners and 19.98% of carnivore cat owners. A comparable trend was observed for sustainability, with 48.15% of vegan dog owners and 39.58% of vegan cat owners rating this aspect with an agreement level of “*strongly agree*”, while only 21.54% of omnivore dog owners and 15.79% of omnivore cat owners rated sustainability with “*strongly agree*”. Omnivore pet owners reported the highest levels of moral disengagement and human supremacy beliefs, whereas vegans showed the lowest values, reflecting their reduced acceptance of speciesism (9, 30). These psychological mechanisms may help to explain why vegan pet owners in our study not only preferred plant-based diets for themselves, but also extended this preference to their pets (30). From a sociological perspective, Gheihman (8) argues that veganism has moved beyond being a marginal animal rights protest and transformed into a “lifestyle movement”, driven by everyday consumption choices and supported by cultural entrepreneurs who have been instrumental in making vegan practices become a mainstream lifestyle (13). Thus, the convergence of our data with prior psychological and sociological research highlights that openness toward non-traditional protein sources in pet nutrition is shaped by a complex interplay of demographic factors, personal dietary identity, and wider cultural shifts (8, 30). These findings are consistent with consumer behavior framework that the owners‘ ethical values are a strong predictor of their personal dietary values and also feeding behavior (31). This pattern may reflect risk-benefit evaluations commonly described in food choice research, where perceived nutritional risks outweigh ethical or sustainable benefits, particularly when feeding obligate carnivores (30).

Insect-based diets were used less frequently and showed lower acceptance among pet owners than vegan alternatives. For example, 3.17% of dog owners fed their animals insect-based dry food daily, only 1.43% used vegetarian and 5.91% plant-based dry food. This contrasts with the survey-based acceptance data from the United Kingdom, where more than half of dog owners expressed a generally positive attitude toward insect-based dog food, even though actual purchase or feeding behavior was not assessed and was assumed to be considerably lower (32). Studies from different regions, including Northern Ireland, Italy and Türkiye, have demonstrated that pet feeding practices, animal health and welfare vary considerably depending on socio-cultural context (33–35). A similar attitude-behavior gap was identified in that study, in which perceived benefits and social norms strongly influenced behavioral intention (32). In contrast to dog owners, among cat owners, insect-based wet food (1.59%) was also the most common alternative, whereas vegan and vegetarian options played a negligible role. This actual feeding behavior contrasts with the owners' stated attitudes: 25.66% of dog owners and 20.08% of cat owners categorically rejected an insect-based diet, while 48.53% and 59.24%, respectively did not consider a vegan diet for their pets. This difference appears to stem from the perception that insect proteins, though unfamiliar, are more biologically appropriate and better aligned with the species-specific dietary needs of dogs and cats (32). In contrast, plant-based diets were widely regarded as potentially harmful (27.80% for dogs; 35.19% for cats), reflecting concerns about their nutritional adequacy, particularly in obligate carnivores like cats, which require nutrients found primarily in animal tissue (10). Owners who were open to alternative proteins cited health and food intolerances as the main reasons for feeding insect- or plant-based diets. Nonetheless, widespread reservations persist, particularly regarding the novelty of insect protein and the adequacy of vegan diets, underscoring the need for more evidence-based communication on the nutritional safety and suitability of such feeding strategies (36).

The survey further reveals a disparity between the recognized importance of diet for pet health and the sources from which owners obtain nutritional information. Although most respondents, 78.45% of the dog owners and 78.73% of the cat owners, indicated that their pets' health was the most important factor in food selection, veterinary advice was only cited by 14.11% of participants as a reason for food choice, while online recommendations (22.95%) were much more influential. This is comparatively low compared with a previous study where 42.58% of pet owners reported considering veterinary recommendations when selecting pet food (37). Despite pet owners overwhelmingly prioritizing their animal's health in food selection, the relatively low reliance on veterinary nutritional guidance compared with online sources highlights a communication gap and underscores the need for veterinarians to more actively provide evidence-based diet information to align owner behavior with professional recommendations (38). In consumer behavior terms, this pattern suggests that trust in information sources plays an important role in shaping feeding decisions, with informal digital sources potentially competing with professional veterinary guidance.

The environmental implications of pet nutrition were acknowledged by a subset of the respondents (dogs: 13.08%; cats: 17.89%) but were overall ranked as less important than health-related factors. This may reflect a lack of awareness about the ecological impact of pet food, which contributes to greenhouse gas emissions and land use (20). These observations are consistent with various studies on human consumption behavior with regard to food. It has been demonstrated that while most consumers have limited knowledge of the environmental impacts of various foods, those with higher levels of knowledge and environmental awareness are more inclined to adopt more sustainable consumption practices (39). Another study demonstrated that demographic factors significantly influence the purchasing behavior of food products labeled with carbon footprint information. Overall, the findings highlight the complexity of consumer behavior, indicating that individuals with greater environmental knowledge and awareness are more likely to consider environmental aspects when making purchasing decisions (40). These findings indicate that consumer choices are influenced by environmental considerations, although such factors often play a secondary role compared to intrinsic quality cues such as health and palatability (41).

Despite confirmed food intolerances, most affected dogs (77.91%) and cats (88.5 %) were still fed meat-based diets. Alternative protein sources such as plant-based (7.98% in dogs) or insect-based diets (7.36% in dogs) were used rarely. This suggests that in the case of intolerance, most pet owners are more likely to resort to other animal protein sources or hydrolyzed diets (42). A clearer adjustment was seen in the choice of carbohydrates: sweet potatoes were significantly more often used in food intolerant dogs (24.90%) compared to those with no food intolerance (12.55%). In cats this shift was not observed.

Overall, these findings highlight that feeding decisions must be interpreted within both a biological and socio-cultural framework, as species-specific nutritional constraints and owner-related factors jointly shape pet feeding practices.

Several limitations of this study should be acknowledged. The sample was highly unbalanced, with a predominance of dog owners, female participants and respondents from Germany. This limits the generalizability of the findings and may introduce sampling bias, as the observed patterns may not fully reflect the broader population of pet owners. In particular, the small proportion of cat owners restricts the validity of species-based comparisons. Differences observed between dog and cat owners should therefore be interpreted with caution, as they may partly reflect unequal group sizes rather than true species-specific effects. The sample also showed a strong predominance of female participants. It remains unclear whether this reflects the actual gender distribution of pet owners or is due to sampling bias related to the recruitment strategy. This imbalance may have affected the observed results.

## 5 Conclusion

This study provides a comprehensive overview of current feeding practices among dog and cat owners, focusing on the acceptance of alternative protein sources and a motivation of pet owners to select the diet. While traditional meat-based diets continue to dominate, a finding of this large-scale survey is that the predominant desire for “higher quality” and “more meat” products remains the leading driver of purchasing decisions. From an ecological perspective, this preference is often associated with a higher environmental footprint, as it can incentivise the prioritization and marketing of resource-intensive, human-edible products for pet food. This is also a noteworthy observation in the context of the current discussions about reducing the use of animal resources.

The data further indicate that rational, evidence-based decision-making is unevenly distributed across pet owner groups. Many pet owners approach nutrition primarily from an anthropocentric welfare perspective, often without deeper nutritional or environmental literacy. In contrast, vegan and vegetarian owners tend to reflect more critically on the ethical and ecological implications of feeding choices and seek more information. This divergence suggests that feeding behavior is not merely shaped by knowledge or access to products, but fundamentally different cognitive and ethical frameworks guiding how owners think about and act regarding their animal's diets.

Overall, the findings highlight the need for targeted education and communication strategies that strengthen rational, evidence-based understanding of species-appropriate and sustainable nutrition. Veterinary professionals play a key role in this context, as they can help to close the gap between owners' ethical values, nutritional adequacy, and ecological responsibility. Future research should therefore focus not only on the physiological suitability of alternative diets, but also on the aspects that influence feeding behavior and its environmental consequences.

## Data Availability

The raw data supporting the conclusions of this article will be made available by the authors, without undue reservation.
