# Supplementary material for: Pet food choices in transition: how owner demographics and diets influence pet food selection and the acceptance of alternative protein sources in pet feeding
Source: Front Vet Sci. 2026 May 15;13:1836864. doi: 10.3389/fvets.2026.1836864 (PMC13218970; doi:10.3389/fvets.2026.1836864)
Supplement: Supplementary file 1 [file Data_Sheet_1.pdf]

Welcome to the survey on feeding practices in our pets. At the Institute for Animal Nutrition from the University of Veterinary Medicine Hannover, Foundation, we would like to investigate how our dogs and cats are fed today and how developments in feeding practices are likely to be in the future.

Answering the following questions will take about 6 - 8 minutes.

Thank you very much for taking the time!

Section A: General Information

A1. In which country do you and your pet(s) live?

A2. Please state your own gender:

- Male ☐
- Female ☐
- Other ☐
- Prefer not to answer ☐

A3. Please state your age:

- <21 ☐
- 21-30 ☐
- 31-40 ☐
- 41-50 ☐
- 51-60 ☐
- 61-70 ☐
- >70 ☐
- No specification ☐

A4. What is your profession?

A5. How would you describe your personal diet?

- Vegan (no animal products) ☐
- Ovo-Lacto-Vegetarian (vegetable products and milk, egg and honey products) ☐
- Mixed diet (plant and animal products) ☐
- No specification ☐

Section B: General pet information

B1. What kind of pet do you have? (If you own a dog and a cat, please use a separate questionnaire for each pet.)

- Dog ☐
- Cat ☐

B2. What breed is your dog?

- [ ] Australian Shepherd
- [ ] Beagle
- [ ] Bernese Mountain Dog
- [ ] Border Collie
- [ ] German Boxer
- [ ] Chihuahua
- [ ] Collie
- [ ] Dachshund
- [ ] Dalmatian
- [ ] German Wirehaired
- [ ] Great Dane
- [ ] German Shorthair
- [ ] German Longhair
- [ ] German Shepherd
- [ ] French Bulldog
- [ ] Golden Retriver
- [ ] Havanese
- [ ] Jack Russel Terrier
- [ ] Small Munsterlander
- [ ] Labrador Retriever
- [ ] Magyar Viszla
- [ ] Maltese
- [ ] Mixed Breed
- [ ] Pug
- [ ] Poodle
- [ ] Rhodesian Ridgeback
- [ ] Rottweiler
- [ ] Schnauzer
- [ ] Shetland Sheepdog
- [ ] Whippet
- [ ] Yorkshire Terrier

B3. What breed is your cat?

- Bengal Cat ☐
- British Shorthair ☐
- European Shorthair ☐
- Birman ☐
- Mixed Breed ☐
- Main Coon ☐
- Norwegian Forest Cat ☐
- Persian Cat ☐
- Ragdoll ☐
- Siamese Cat ☐
- Other ☐

Other

[ ]

B4. How old is your dog/cat in years and months? (If unknown, please estimate approximate age)

Years [ ]

Months [ ]

B5. How much does your dog/cat weigh (in kilograms)?

bodyweight (kg) [ ]

B6. What sex is your dog/cat?

- Male ☐
- Male neutered ☐
- Female ☐
- Female spayed ☐
- I don`t know ☐

B7. Does your animal suffer from adverse feed reactions or even allergies to feed?

- No ☐
- I suspect it, but no diagnostics have been done. ☐
- Yes and this was proven by a blood test. ☐
- Yes and this was proven by an elimination diet. ☐

Section C: Feeding

C1. What kind of feed do you currently feed and how often?

	Daily	Frequently (one to two times a week)	Rarely (one to two times a month)	Never
Wet food (meat based)	☐	☐	☐	☐
Wet food (insect based)	☐	☐	☐	☐
Wet food (vegetarian)	☐	☐	☐	☐
Wet food (vegan)	☐	☐	☐	☐
Dry food (meat based)	☐	☐	☐	☐
Dry food (insect based)	☐	☐	☐	☐
Dry food (vegetarian)	☐	☐	☐	☐
Dry food (vegan)	☐	☐	☐	☐
Homemade diet	☐	☐	☐	☐
Raw diet (BARF)	☐	☐	☐	☐

C2. If you feed your pet an insect based diet, how long have you been feeding it?

- <1 year ☐
- 1-2 years ☐
- 3-4 years ☐
- 5-6 years ☐
- >6 years ☐
- I don't feed an insect based diet. ☐

C3. If you feed your pet a vegan diet, how long have you been feeding it?

- <1 year ☐
- 1-2 years ☐
- 3-4 years ☐
- 5-6 years ☐
- >6 years ☐
- I don't feed a vegan diet ☐

C4. On which protein source is your currently mainly used feed based? (Please have a look at the declaration, which can usually be found on the back of the feed.)

- Meat and/or slaughter by-products (e.g. beef or chicken hearts) ☐
- Vegetarian protein sources (e.g. eggs and dairy products) ☐
- Vegetable protein sources (e.g. peas and lentils) ☐
- Insects (e.g. Hermetia Illucens) ☐
- Fish (e.g. Salmon) ☐
- Other ☐

Other

[ ]

C5. On which carbohydrate source is your currently mainly used feed based? (Please have a look at the declaration, which can usually be found on the back of the feed.)

- Rice ☐
- Wheat ☐
- Potato ☐
- Sweet potato ☐
- Maize ☐
- Manioc ☐
- Tapioca ☐
- Barley ☐
- Quinoa ☐
- Other ☐

Other

[ ]

C6. Do you feed your pet snacks, chews or treats?

- Several times a day ☐
- Once a day ☐
- Several times a week ☐
- Several times a month ☐
- Never ☐

C7. Which kind of snacks, chews or treats do you feed?

- Vegan snacks ☐
- Vegetarian snacks ☐
- Meat based snacks ☐
- Insect based snacks ☐

Section D: Food choice

D1. How did you decide on the current diet for your pet?

- On the advice of my veterinarian ☐
- On the advice of the breeder ☐
- On the advice of another pet owner ☐
- Through information on the packaging of pet food ☐
- Through information in books or magazines ☐
- Through information on the internet ☐
- Through advice in the pet shop ☐
- Other ☐

Other

D2. Where do you normally buy your pet food?

- Supermarket ☐
- Online ☐
- Veterinary practice ☐
- Pet store ☐
- Manufacturer ☐
- Butcher ☐
- Other ☐

Other

D3. What is particularly important to you when choosing feed with regard to the use of meat?

1 (very important) – 5 (not important)

	1	2	3	4	5
High meat content	☐	☐	☐	☐	☐
Low meat content	☐	☐	☐	☐	☐
High lean meat content	☐	☐	☐	☐	☐
Low lean meat content	☐	☐	☐	☐	☐
Use of animal by-products	☐	☐	☐	☐	☐
No use of animal by-products	☐	☐	☐	☐	☐
Regionality	☐	☐	☐	☐	☐
Organic production	☐	☐	☐	☐	☐

D4. How do you think about the use of grain (e.g. wheat) in petfood?

1 (strongly agree) – 5 (strongly disagree)

	1	2	3	4	5
It is important to me that no grain is used.	☐	☐	☐	☐	☐
It is important to me that grain is used.	☐	☐	☐	☐	☐
I have no preferences with regard to the use of grain.	☐	☐	☐	☐	☐

D5. How important are the following parameters to you when assessing the quality of a feed?

1 (very important) – 5 (not important)

	1	2	3	4	5
Meat content	☐	☐	☐	☐	☐
Organic quality	☐	☐	☐	☐	☐
Price	☐	☐	☐	☐	☐
Grain content	☐	☐	☐	☐	☐
Smell and look	☐	☐	☐	☐	☐
Protein content	☐	☐	☐	☐	☐
Content of vegetable ingredients	☐	☐	☐	☐	☐

D6. What aspects do you pay attention to when feeding meat-based feed?

1 (Applies fully) – 5 (Not at all)

	1	2	3	4	5
I try to pay attention to animal welfare aspects of farm animals.	☐	☐	☐	☐	☐
I make sure to feed a sustainable food as much as possible.	☐	☐	☐	☐	☐
For me, the focus is on the health of my animal.	☐	☐	☐	☐	☐
For me, an organic production is important.	☐	☐	☐	☐	☐

D7. Should you prepare the food for your pet yourself, where did you get the "recipe" from?

- Own feeling ☐
- Internet ☐
- Specialist literature ☐
- Nutritional consultant (not veterinarian) ☐
- Veterinarian ☐
- Specialized veterinarian for animal nutrition ☐
- I use conventional feed ☐

Section E: Alternative protein sources

E1. What do you think about feeding a meat-based diet?

1 (Applies fully) – 5 (Not at all)

	1	2	3	4	5
I think a meat-based diet is species-appropriate.	☐	☐	☐	☐	☐
I think that a meat-based diet does contain all thenutrients needed.	☐	☐	☐	☐	☐
I am afraid that a meat based diet is harmful to my pet's health.	☐	☐	☐	☐	☐
I think that meat-based diet is not to my animal's liking.	☐	☐	☐	☐	☐
I think that my animal does tolerate a meat-based diet well.	☐	☐	☐	☐	☐
I think a meat-based diet is expensive.	☐	☐	☐	☐	☐

E2. What do you think about feeding a vegan diet?

1 (Applies fully) – 5 (Not at all)

	1	2	3	4	5
I think that a vegan diet is species-appropriate.	☐	☐	☐	☐	☐
I think that a vegan diet contains all the nutrients needed.	☐	☐	☐	☐	☐
I am afraid that a vegan diet is harmful to my pet's health.	☐	☐	☐	☐	☐
I think that my pet would't like a vegan diet.	☐	☐	☐	☐	☐
I think that my animal does tolerate vegan food well.	☐	☐	☐	☐	☐
I think vegan food is expensive.	☐	☐	☐	☐	☐

E3. What do you think about feeding an insect-based diet?

1 (Applies fully) – 5 (Not at all)

	1	2	3	4	5
I think that a food containing insects is species-appropriate.	☐	☐	☐	☐	☐
I think that a food containing insects does contain all the nutrients needed.	☐	☐	☐	☐	☐
I am afraid that a food containing insects is harmful to my pet's health.	☐	☐	☐	☐	☐
I think that a food containing insects is not to my animal's liking.	☐	☐	☐	☐	☐
I think that my animal does tolerate insect food well.	☐	☐	☐	☐	☐
I think an insect food is expensive.	☐	☐	☐	☐	☐

E4. Would you feed a vegan diet?

- Yes ☐
- No ☐
- Maybe ☐

E5. Would you feed a diet based on insects?

- Yes ☐
- No ☐
- Maybe ☐

E6. Assuming a vegan diet is containing all the nutrients your pet needs, would you feed it permanently?

- Yes ☐
- No ☐
- Maybe ☐

E7. Assuming an insect-based diet is containing all the nutrients your pet needs, would you feed it permanently?

- Yes ☐
- No ☐
- Maybe ☐

E8. Assuming that your dog / cat would like a vegan diet very much, would you feed it permanently?

- Yes ☐
- No ☐
- Maybe ☐

E9. Assuming that your pet would like an insect-based food very much, would you feed it permanently?

- Yes ☐
- No ☐
- Maybe ☐

E10. What would be reasons for you to buy a vegan food?

1 (Applies fully) – 5 (Not at all)

	1	2	3	4	5
Animal welfare of farm animals	☐	☐	☐	☐	☐
Adverse food reactions of my pet	☐	☐	☐	☐	☐
Sustainability	☐	☐	☐	☐	☐
Health of my pet	☐	☐	☐	☐	☐
Price	☐	☐	☐	☐	☐

E11. What would be reasons for you to buy a insect based food?

1 (Applies fully) – 5 (Not at all)

	1	2	3	4	5
Animal welfare of farm animals	☐	☐	☐	☐	☐
Adverse food reactions of my pet	☐	☐	☐	☐	☐
Sustainability	☐	☐	☐	☐	☐
Health of my pet	☐	☐	☐	☐	☐
Price	☐	☐	☐	☐	☐

E12. Would you use alternative protein sources (plant or insect based) if you had more information about the adequacy of requirements for your animal?

- Yes ☐
- No ☐
- Maybe ☐

E13. How do you think dog and cat food will change in the future?

1 (Applies fully) – 5 (Not at all)

	1	2	3	4	5
Lower meat content	☐	☐	☐	☐	☐
Lower quality meat	☐	☐	☐	☐	☐
Higher quality meat	☐	☐	☐	☐	☐
Higher meat content	☐	☐	☐	☐	☐
Less grain	☐	☐	☐	☐	☐
More grain	☐	☐	☐	☐	☐
More frequent use of vegetable protein source	☐	☐	☐	☐	☐
More frequent use of insects as a source of protein	☐	☐	☐	☐	☐
Higher proportion of organic quality	☐	☐	☐	☐	☐

E14. How do you wish dog and cat food will change in the future?

	1	2	3	4	5
Lower meat content	☐	☐	☐	☐	☐
Forced use of animal by-products	☐	☐	☐	☐	☐
Higher quality meat	☐	☐	☐	☐	☐
Higher meat content	☐	☐	☐	☐	☐
Less grain	☐	☐	☐	☐	☐
More grain	☐	☐	☐	☐	☐
More frequent use of vegetable protein source	☐	☐	☐	☐	☐
More frequent use of insects as a source of protein	☐	☐	☐	☐	☐
Higher proportion of organic quality	☐	☐	☐	☐	☐

Thank you very much for your participation!
