# Supplementary material for: Pet food choices in transition: how owner demographics and diets influence pet food selection and the acceptance of alternative protein sources in pet feeding
Source: Front Vet Sci. 2026 May 15;13:1836864. doi: 10.3389/fvets.2026.1836864 (PMC13218970; doi:10.3389/fvets.2026.1836864)
Supplement: Supplementary file 2 [file Data_Sheet_2.pdf]

Herzlich Willkommen zur Umfrage zu den Fütterungsgewohnheiten bei unseren Haustieren. Wir von dem Institut für Tierernährung der Stiftung Tierärztliche Hochschule Hannover möchten erforschen, wie unsere Hunde und Katzen heutzutage gefüttert werden und wie sich Entwicklungen in der Fütterungspraxis in Zukunft vermutlich darstellen werden.

Die Beantwortung der folgenden Fragen wird ca. 6 - 8 Minuten in Anspruch nehmen.

Vielen Dank, dass Sie sich die Zeit nehmen!

Teil A: Allgemeine Informationen

A1. In welchem Land leben Sie und Ihr/Ihre Haustier/Haustiere?

A2. Bitte geben Sie Ihr eigenes Geschlecht an.

- Männlich ☐
- Weiblich ☐
- Divers ☐
- Keine Angabe ☐
- Sonstiges ☐

Sonstiges

A3. Bitte geben Sie ihr Alter an:

- < 21 ☐
- 21 - 30 ☐
- 31 - 40 ☐
- 41 - 50 ☐
- 51 - 60 ☐
- 61 - 70 ☐
- > 70 ☐
- Keine Angabe ☐

A4. Welchen Beruf üben Sie aus?

A5. Wie ernähren Sie sich?

- Vegan (keine tierischen Produkte) ☐
- Ovo - Lacto - Vegetarisch (Pflanzliche Produkte sowie Milch-, Eier- und Honigerzeugnisse) ☐
- Mischkost (pflanzliche und tierische Produkte) ☐
- Keine Angabe ☐

Teil B: Haustierinformationen

B1. Welches der genannten Haustiere besitzen Sie? (Sollten Sie sowohl einen Hund, als auch eine Katze besitzen, füllen Sie je weils einen Fragebogen aus.)

- Hund ☐
- Katze ☐

B2. Welcher Rasse gehört Ihr Hund an?

- Australian Shepherd ☐
- Beagle ☐
- Berner Sennenhund ☐
- Border Collie ☐
- Boxer ☐
- Chihuahua ☐
- Collie ☐
- Dackel ☐
- Dalmatiner ☐
- Deutsch Drahthaar ☐
- Deutsche Dogge ☐
- Deutsch Kurzhaar ☐
- Deutsch Langhaar ☐
- Deutscher Schäferhund ☐
- Französische Bulldogge ☐
- Golden Retriver ☐
- Havaneser ☐
- Jack Russel Terrier ☐
- Kleiner Münsterländer ☐
- Labrador ☐
- Magyar Vizsla ☐
- Malteser ☐
- Mischling ☐
- Mops ☐
- Pudel ☐
- Rhodesian Ridgeback ☐
- Rottweiler ☐
- Schnauzer ☐
- Shetland Sheepdog ☐
- Whippet ☐
- Yorkshire Terrier ☐

B3. Welcher Rasse gehört Ihre Katze an?

- Bengalkatze ☐
- Britisch Kurzhaar ☐
- Europäisch Kurzhaar ☐
- Heilige Birma ☐
- Maine Coon ☐
- Mischling ☐
- Norwegische Waldkatze ☐
- Perserkatze ☐
- Ragdoll ☐
- Siamkatze ☐
- Sonstiges ☐

Sonstiges

[ ]

B4. Wie alt ist Ihr Hund oder Ihre Katze in Jahren und Monaten? (Falls unbekannt, schätzen Sie bitte das ungefähre Alter)

Jahre [ ]

Monate [ ]

B5. Wieviel wiegt Ihr Hund / Ihre Katze (in Kilogramm) ?

Körpergewicht in Kilogramm [ ]

B6. Welches Geschlecht hat Ihr Hund / Ihre Katze?

- Männlich unkastriert ☐
- Männlich kastriert ☐
- Weiblich unkastriert ☐
- Weiblich kastriert ☐
- Ich weiß es nicht ☐

B7. Leidet Ihr Tier unter Unverträglichkeitsreaktionen oder gar Allergien gegenüber Futtermitteln?

- [ ] Nein
- [ ] Ich vermute es, aber es wurde keine Diagnostik gemacht.
- [ ] Ja und dieses wurde durch einen Bluttest nachgewiesen.
- [ ] Ja und dieses wurde durch eine Ausschlussdiät nachgewiesen.

Teil C: Fütterung

C1. Welche Art von Futter füttern Sie derzeit wie häufig?

	Täglich	Häufig (ein bis zwei Mal die Woche)	Selten (ein bis zwei Mal im Monat)	Nie
Nassfutter (fleischbasiert)	☐	☐	☐	☐
Nassfutter (insektenbasiert)	☐	☐	☐	☐
Nassfutter (vegetarisch)	☐	☐	☐	☐
Nassfutter (Vegan)	☐	☐	☐	☐
Trockenfutter (fleischbasiert)	☐	☐	☐	☐
Trockenfutter (insektenbasiert)	☐	☐	☐	☐
Trockenfutter (vegetarisch)	☐	☐	☐	☐
Trockenfutter (vegan)	☐	☐	☐	☐
Selbstgekochtes Futter	☐	☐	☐	☐
Rohkost-Diät (BARF)	☐	☐	☐	☐
Sonstiges	☐	☐	☐	☐

C2. Sollten Sie Ihrem Haustier eine vegane Diät füttern, wie lange füttern Sie diese schon?

- [ ] < 1 Jahr
- [ ] 1 - 2 Jahre
- [ ] 3 - 4 Jahre
- [ ] 5 - 6 Jahre
- [ ] > 6 Jahre
- [ ] Ich füttere kein veganes Futter

C3. Sollten Sie Ihrem Haustier eine insektenbasierte Diät füttern, wie lange füttern Sie diese schon?

- < 1 Jahr ☐
- 1 - 2 Jahre ☐
- 3 - 4 Jahre ☐
- 5 - 6 Jahre ☐
- > 6 Jahre ☐
- Ich füttere kein insektenbasiertes Futter ☐

C4. Auf welcher Proteinquelle basiert Ihr zurzeit hauptsächlich verwendetes Futter? (Schauen Sie dazu bitte auf die Deklaration des Futters, diese befindet sich in der Regel auf der Rückseite des Gebindes.)

- Fleisch und/oder Schlachtnebenprodukte (z.B. Rindfleisch oder Hühnerherzen) ☐
- Vegetarische Proteinquellen (z.B. Eier und Milchprodukte) ☐
- Pflanzliche Proteinquellen (z.B. Erbsen und Linsen) ☐
- Insekten (z.B. Hermetia Illucens) ☐
- Fisch (z.B. Lachs) ☐

C5. Auf welcher Kohlenhydratquelle basiert Ihr zurzeit hauptsächlich verwendetes Futter? (Schauen Sie dazu bitte auf die Deklaration des Futters, diese befindet sich in der Regel auf der Rückseite des Gebindes.)

- Reis ☐
- Weizen ☐
- Kartoffel ☐
- Süßkartoffel ☐
- Mais ☐
- Maniok ☐
- Tapioka ☐
- Gerste ☐
- Quinoa ☐
- Andere ☐

C6. Füttern Sie Ihrem Haustier Snacks, Kauartikel oder Leckerlis?

- Mehrmals am Tag ☐
- Einmal am Tag ☐
- Ein paar Mal pro Woche ☐
- Ein paar Mal im Monat ☐
- Weniger als zweimal im Monat ☐
- Nie ☐

C7. Welche Art von Snacks, Kauartikeln oder Leckerlis verwenden Sie? (Mehrfachauswahl)

- Vegane Snacks ☐
- Vegetarische Snacks ☐
- Fleischhaltige Snacks ☐
- Insektenhaltige Snacks ☐

Teil D: Futterwahl

D1. Wie haben Sie sich für Ihr aktuelles Futter entschieden?

- Auf Anraten meines Tierarztes ☐
- Auf Anraten des Züchters/Tierheims ☐
- Auf Anraten eines anderen Tierbesitzers ☐
- Durch Informationen auf der Verpackung von Tiernahrung ☐
- Durch Informationen in Büchern/Zeitschriften ☐
- Durch Informationen im Internet ☐
- Durch Beratung im Fachhandel ☐
- Andere ☐

D2. Wo kaufen Sie normalerweise Ihr Tierfutter? (Mehrfachauswahl)

- Supermarkt ☐
- Online ☐
- Tierarztpraxis ☐
- Zoofachmarkt ☐
- Hersteller ☐
- Metzger ☐

Andere ☐

D3. Auf welche Aspekte achten Sie bei der Fütterung von fleischhaltigem Futter?

1 (trifft voll zu) – 5 (trifft gar nicht zu)

	1	2	3	4	5
Ich versuche auf Tierwohlaspekte von Nutztieren zu achten.	☐	☐	☐	☐	☐
Ich achte darauf ein möglichst nachhaltiges Futter zu verfüttern.	☐	☐	☐	☐	☐
Für mich steht die Gesundheit meines Tieres im Fokus.	☐	☐	☐	☐	☐
Ich bevorzuge eine ökologische Erzeugung.	☐	☐	☐	☐	☐

D4. Wie wichtig sind Ihnen folgende Parameter bei der Beurteilung der Qualität eines Futters?

1 (sehr wichtig) - 5 (unwichtig)

	1	2	3	4	5
Fleischgehalt	☐	☐	☐	☐	☐
Bioqualität	☐	☐	☐	☐	☐
Preis	☐	☐	☐	☐	☐
Getreideanteil	☐	☐	☐	☐	☐
Geruch und Aussehen	☐	☐	☐	☐	☐
Proteingehalt	☐	☐	☐	☐	☐
Anteil pflanzlicher Komponenten	☐	☐	☐	☐	☐

D5. Sollten Sie das Futter für Ihr Haustier selbst zubereiten, woher haben Sie das „Rezept“?

- Eigenes Empfinden ☐
- Internet ☐
- Fachliteratur ☐
- Ernährungsberater:in (nicht Tierarzt/Tierärztin) ☐
- Tierarzt / Tierärztin ☐
- Fachtierarzt für Tierernährung ☐
- Ich nutze ein fertiges Futter ☐

D6. Was ist Ihnen bei der Futterauswahl im Hinblick auf die Verwendung von Fleisch besonders wichtig?

1 (sehr wichtig) - 5 (unwichtig)

	1	2	3	4	5
Hoher Fleischanteil	☐	☐	☐	☐	☐
Niedriger Fleischanteil	☐	☐	☐	☐	☐
Hoher Muskelfleischanteil	☐	☐	☐	☐	☐
Niedriger Muskelfleischanteil	☐	☐	☐	☐	☐
Verwendung von tierischen Nebenerzeugnissen	☐	☐	☐	☐	☐
Keine Verwendung von tierischen Nebenerzeugnissen	☐	☐	☐	☐	☐
Regionalität	☐	☐	☐	☐	☐
Ökologische Erzeugung	☐	☐	☐	☐	☐

D7. Was halten Sie von der Verwendung von Getreide (z. B. Weizen) im Hundefutter? 1 (stimme voll zu) - 5 (stimme überhaupt nicht zu)

	1	2	3	4	5
Es ist mir wichtig, dass kein Getreide genutzt wird.	☐	☐	☐	☐	☐
Es ist mir wichtig, dass Getreide genutzt wird.	☐	☐	☐	☐	☐
Ich habe keine Präferenzen in Bezug auf die Nutzung von Getreide.	☐	☐	☐	☐	☐

Teil E: Alternative Proteinquellen

E1. Wie stehen Sie zur Fütterung eines fleischhaltigen Futters? 1 (trifft voll zu) - 5 (trifft gar nicht zu)

	1	2	3	4	5
Ich denke, dass ein fleischhaltiges Futter artgerecht ist.	☐	☐	☐	☐	☐
Ich denke, dass ein fleischhaltiges Futter alle benötigten Nährstoffe enthält.	☐	☐	☐	☐	☐
Ich denke, dass ein fleischhaltiges Futter gesundheitsschädlich für meinen Hund ist.	☐	☐	☐	☐	☐
Ich denke, dass ein fleischhaltiges Futter meinem Tier nicht schmeckt.	☐	☐	☐	☐	☐
Ich denke, dass mein Tier ein fleischhaltiges Futter nicht gut verträgt.	☐	☐	☐	☐	☐
Ich denke, dass ein fleischhaltiges Futter teuer ist.	☐	☐	☐	☐	☐

E2. Wie stehen Sie zur Fütterung eines veganen Futters?

1 (trifft voll zu) – 5 (trifft gar nicht zu)

	1	2	3	4	5
Ich denke, dass ein veganes Futter artgerecht ist.	☐	☐	☐	☐	☐
Ich denke, dass ein veganes Futter alle benötigten Nährstoffe enthält.	☐	☐	☐	☐	☐
Ich habe Angst, dass ein veganes Futter gesundheitsschädlich für meinen Hund ist.	☐	☐	☐	☐	☐
Ich denke, dass ein veganes Futter meinem Tier nicht schmeckt.	☐	☐	☐	☐	☐
Ich denke, dass mein Tier ein veganes Futter gut verträgt.	☐	☐	☐	☐	☐
Ich denke, dass ein veganes Futter teuer ist.	☐	☐	☐	☐	☐

E3. Wie stehen Sie zur Fütterung eines insektenhaltigen Futters?

1 (trifft voll zu) - 5 (trifft gar nicht zu)

	1	2	3	4	5
Ich denke, dass ein insektenhaltiges Futter artgerecht ist.	☐	☐	☐	☐	☐
Ich denke, dass ein insektenhaltiges Futter alle benötigten Nährstoffe enthält.	☐	☐	☐	☐	☐
Ich habe Angst, dass ein insektenhaltiges Futter gesundheitsschädlich für meine Tier ist.	☐	☐	☐	☐	☐
Ich denke, dass ein insektenhaltiges Futter meinem Tier nicht schmeckt.	☐	☐	☐	☐	☐
Ich denke, dass mein Tier ein insektenhaltiges Futter gut verträgt.	☐	☐	☐	☐	☐
Ich denke, dass ein insektenhaltiges Futter teuer ist.	☐	☐	☐	☐	☐

E4. Würden Sie alternative Proteinquellen (pflanzlich oder insektenbasiert) verwenden, wenn Ihnen mehr Informationen über die Bedarfsgerechtigkeit für Ihr Tier zur Verfügung stehen würden?

- Ja ☐
- Nein ☐
- Vielleicht ☐

E5. Würden Sie ein veganes Futter füttern?

- Ja ☐
- Nein ☐
- Vielleicht ☐

E6. Würden Sie ein Futter auf Grundlage von Insekten füttern?

- Ja ☐
- Nein ☐
- Vielleicht ☐

E7. Angenommen, ein veganes Futter würde alle Nährstoffe enthalten, die Ihr Haustier benötigt, würden Sie dieses dann dauerhaft füttern?

- Ja ☐
- Nein ☐
- Vielleicht ☐

E8. Angenommen, ein insektenbasiertes Futter würde alle Nährstoffe enthalten, die Ihr Haustier benötigt, würden Sie dieses dann dauerhaft füttern?

- Ja ☐
- Nein ☐
- Vielleicht ☐

E9. Angenommen, ein veganes Futter würde Ihrem Haustier sehr gut schmecken, würden Sie dieses dann dauerhaft füttern?

- Ja ☐
- Nein ☐
- Vielleicht ☐

E10. Angenommen, ein insektenbasiertes Futter würde Ihrem Haustier sehr gut schmecken, würden Sie dieses dann dauerhaft füttern?

- Ja ☐
- Nein ☐
- Vielleicht ☐

E11. Was wären Gründe für Sie, ein veganes Futter zu kaufen?

1 (trifft voll zu) – 5 (trifft gar nicht zu)

	1	2	3	4	5
Das Wohlergehen von Nutztieren	☐	☐	☐	☐	☐
Gesundheit meines Haustieres	☐	☐	☐	☐	☐
Nachhaltigkeit	☐	☐	☐	☐	☐
Unverträglichkeitsreaktionen meines Haustieres auf andere Futter	☐	☐	☐	☐	☐
Preis	☐	☐	☐	☐	☐

E12. Was wären Gründe für Sie, ein insektenbasiertes Futter zu kaufen?

1 (trifft voll zu) – 5 (trifft gar nicht zu)

	1	2	3	4	5
Das Wohlergehen von Nutztieren	☐	☐	☐	☐	☐
Gesundheit meines Haustieres	☐	☐	☐	☐	☐
Nachhaltigkeit	☐	☐	☐	☐	☐
Unverträglichkeitsreaktionen meines Haustieres auf andere Futter	☐	☐	☐	☐	☐
Preis	☐	☐	☐	☐	☐

E13. Wie wird sich Ihrer Meinung nach das Futter für Hunde und Katzen in Zukunft verändern?

1 (trifft voll zu) – 5 (trifft gar nicht zu)

	1	2	3	4	5
Geringerer Fleischanteil	☐	☐	☐	☐	☐
Höherer Fleischanteil	☐	☐	☐	☐	☐
Qualitativ hochwertigeres Fleisch	☐	☐	☐	☐	☐
Qualitativ minderwertigeres Fleisch	☐	☐	☐	☐	☐
Weniger Getreide	☐	☐	☐	☐	☐
Mehr Getreide	☐	☐	☐	☐	☐
Häufigere Nutzung pflanzlicher Proteinquelle	☐	☐	☐	☐	☐
Häufigere Nutzung von Insekten als Proteinquelle	☐	☐	☐	☐	☐
Höherer Anteil an Bioqualität	☐	☐	☐	☐	☐

E14. Welche Art von Veränderung des Futters für Hunde und Katzen wünschen Sie sich in Zukunft?

1 (trifft voll zu) – 5 (trifft gar nicht zu)

	1	2	3	4	5
Geringerer Fleischanteil	☐	☐	☐	☐	☐
Höherer Fleischanteil	☐	☐	☐	☐	☐
Qualitativ hochwertigeres Fleisch	☐	☐	☐	☐	☐
Forcierte Verwendung von Schlachtnebenprodukten	☐	☐	☐	☐	☐
Weniger Getreide	☐	☐	☐	☐	☐
Mehr Getreide	☐	☐	☐	☐	☐
Häufigere Nutzung pflanzlicher Proteinquelle	☐	☐	☐	☐	☐
Häufigere Nutzung von Insekten als Proteinquelle	☐	☐	☐	☐	☐
Höherer Anteil an Bioqualität	☐	☐	☐	☐	☐

Danke für Ihre Teilnahme!
